# Process Evaluation of a Wireless Wearable Continuous Vital Signs Monitoring Intervention in 2 General Hospital Wards: Mixed Methods Study

**DOI:** 10.2196/44061

**Published:** 2023-05-04

**Authors:** Jobbe P L Leenen, Henriëtte J M Rasing, Cor J Kalkman, Lisette Schoonhoven, Gijsbert A Patijn

**Affiliations:** 1 Connected Care Center Isala Zwolle Netherlands; 2 Isala Academy Isala Zwolle Netherlands; 3 Department of Surgery Isala Zwolle Netherlands; 4 Department of Internal Medicine Isala Zwolle Netherlands; 5 Department of Anaesthesiology University Medical Center Utrecht Utrecht Netherlands; 6 University Medical Center Utrecht Julius Center for Health Sciences and Primary Care Utrecht University Utrecht Netherlands; 7 School of Health Sciences Faculty of Environmental and Life Sciences University of Southampton Southampton United Kingdom

**Keywords:** physiological monitoring, implementation science, clinical deterioration, continuous vital sign monitoring, wearable wireless devices, wearables, process evaluation, mixed methods, intervention fidelity

## Abstract

**Background:**

Continuous monitoring of vital signs (CMVS) using wearable wireless sensors is increasingly available to patients in general wards and can improve outcomes and reduce nurse workload. To assess the potential impact of such systems, successful implementation is important. We developed a CMVS intervention and implementation strategy and evaluated its success in 2 general wards.

**Objective:**

We aimed to assess and compare intervention fidelity in 2 wards (internal medicine and general surgery) of a large teaching hospital.

**Methods:**

A mixed methods sequential explanatory design was used. After thorough training and preparation, CMVS was implemented—in parallel with the standard intermittent manual measurements—and executed for 6 months in each ward. Heart rate and respiratory rate were measured using a chest-worn wearable sensor, and vital sign trends were visualized on a digital platform. Trends were routinely assessed and reported each nursing shift without automated alarms. The primary outcome was intervention fidelity, defined as the proportion of written reports and related nurse activities in case of deviating trends comparing early (months 1-2), mid- (months 3-4), and late (months 5-6) implementation periods. Explanatory interviews with nurses were conducted.

**Results:**

The implementation strategy was executed as planned. A total of 358 patients were included, resulting in 45,113 monitored hours during 6142 nurse shifts. In total, 10.3% (37/358) of the sensors were replaced prematurely because of technical failure. Mean intervention fidelity was 70.7% (SD 20.4%) and higher in the surgical ward (73.6%, SD 18.1% vs 64.1%, SD 23.7%; *P*<.001). Fidelity decreased over the implementation period in the internal medicine ward (76%, 57%, and 48% at early, mid-, and late implementation, respectively; *P*<.001) but not significantly in the surgical ward (76% at early implementation vs 74% at midimplementation [*P*=.56] vs 70.7% at late implementation [*P*=.07]). No nursing activities were needed based on vital sign trends for 68.7% (246/358) of the patients. In 174 reports of 31.3% (112/358) of the patients, observed deviating trends led to 101 additional bedside assessments of patients and 73 consultations by physicians. The main themes that emerged during interviews (n=21) included the relative priority of CMVS in nurse work, the importance of nursing assessment, the relatively limited perceived benefits for patient care, and experienced mediocre usability of the technology.

**Conclusions:**

We successfully implemented a system for CMVS at scale in 2 hospital wards, but our results show that intervention fidelity decreased over time, more in the internal medicine ward than in the surgical ward. This decrease appeared to depend on multiple ward-specific factors. Nurses’ perceptions regarding the value and benefits of the intervention varied. Implications for optimal implementation of CMVS include engaging nurses early, seamless integration into electronic health records, and sophisticated decision support tools for vital sign trend interpretation.

## Introduction

### Background

Most adverse events occurring in hospital wards are preceded by a considerable period of changes in vital signs, which are important indicators of clinical deterioration [1]. Monitoring vital signs allows for early detection and timely interventions that may improve outcomes [1-4]. In high-care units with patients who are critically ill, continuous monitoring of vital signs (CMVS) is the norm, whereas in general wards, vital signs are usually monitored intermittently, and interpretation is guided by Early Warning Scores (EWSs) [5-7]. Although the EWS system may facilitate early detection, there are still limitations owing to its intermittent nature and variable compliance [8-10]. Consequently, patients’ condition may deteriorate unnoticed, which can lead to avoidable adverse events, adverse outcomes, and higher costs [11-13].

Given recent technological developments, CMVS using wearable wireless sensors has become available to patients in general wards. Previous studies have shown that these systems can accurately measure vital signs and detect deterioration [14-16]. However, evidence on the effects of these CMVS systems on patient outcomes is scarce [17,18]. This may be related to the fact that the implementation of CMVS at scale remains challenging and requires considerable upfront financial investment by hospital administrations [19,20].

Although many health care professionals acknowledge the potential benefits of CMVS for patient care, several studies have highlighted considerable challenges, such as difficult implementation into existing nursing workflows, poor integration with hospital electronic health record (EHR) systems, and primitive alarm management strategies [21-23]. In addition, monitoring vital sign trends may be challenging for most ward nurses because of a lack of experience with interpreting graphic representations of CMVS trends [15,24,25].

### Objectives

Given these challenges, implementing CMVS in hospital wards is considered a “complex intervention” with many interacting components and the need for behavior change of health care professionals and affecting multiple patient outcomes [26,27]. Successfully scaled implementation in wards is necessary before any possible beneficial effects of CMVS on clinical outcomes can be expected [17,21,28]. Unfortunately, there is only scant knowledge on the facilitators and barriers to CMVS implementation [24,25,29]. We have previously conducted 2 feasibility studies [15,23] and 2 qualitative studies [24,30] that aided in developing and refining our CMVS intervention and an implementation strategy. For this study, an implementation-effectiveness hybrid design was used for the parallel evaluation of the implementation and effectiveness of the intervention [31]. This report focuses on the process evaluation of the implementation, with the primary aim of assessing and comparing intervention fidelity in 2 wards (internal medicine and general surgery). The secondary aims were to assess and compare implementation fidelity, technical fidelity, perceived appropriateness, acceptability, usability, adoption, and feasibility according to nurses. The effectiveness of the intervention will be analyzed and described in a separate paper.

## Methods

### Design

A mixed methods sequential explanatory design [32] was used for an 8-month period in a surgical ward and an internal medicine ward (September 2021-July 2022) of a 1245-bed tertiary teaching hospital in the Netherlands. The study was reported according to the Standards for Quality Improvement Reporting Excellence 2.0 checklist [33].

### Context

The surgical ward consisted of 49 beds, in which patients of gastrointestinal and vascular surgery were admitted. A total of 57.4 full-time equivalent of nurses were employed at the ward. The nurse-to-patient ratio was 1:5 for the day shift, 1:6 for the evening shift, and 1:12 for the night shift. A nurse specialist or junior resident assessed the patients daily during morning rounds.

The internal medicine ward consisted of 48 beds and was divided into 2 teams of nurses based on subspecialties: general internal medicine and gastroenterology. The nurse-to-patient ratio was 1:4 for the day shift, 1:12 for the evening shift, and 1:12 for the night shift. A junior resident assessed the patients during morning rounds.

Patients admitted to the surgical and internal medicine wards were eligible to receive the CMVS intervention (Multimedia Appendix 1). Inclusion criteria were age of ≥18 years, no cognitive impairments, expected hospitalization time of ≥2 days, and ability to speak and read the Dutch language. The exclusion criterion was inability to wear the CMVS sensor because of an allergy. Nurses who were employed at the ward during the study period participated and were eligible for participation in the process evaluation if they had worked with the CMVS system for at least one month during the study period. Nurses temporarily employed from the flex pool were excluded from the study.

### Intervention

In addition to standard care, patients included in the study were monitored using the Conformité Européene–marked Healthdot sensor (Philips Healthcare) and IntelliVue Guardian Solution (IGS) software platform (*Philips Healthcare*). Standard care consisted of intermittent monitoring (at least once daily) through manual measurements performed by the nurse and assessed using the Modified EWS (MEWS) according to the local hospital protocol [34].

The wireless wearable sensor was a water-resistant disposable patch worn on the patient’s chest (Multimedia Appendix 2); it continuously records the heart rate (HR) in beats per minute and respiratory rate (ReR) in respirations per minute both using accelerometry. Previous studies have shown that this sensor is accurate [35,36]. The 2 vital sign measurements are transmitted wirelessly every 5 minutes through a long-range, low-power Internet of Things connection (LoRa; Semtech) to the IGS software. After connecting the sensor to the patient and to the software by scanning the QR code using a separate mobile phone, the software platform with trends was displayed on the computer on wheels and in a mobile app (Multimedia Appendix 3). The battery life of the patch was 14 days, and during the performance of an electrocardiogram, computer tomography, or magnetic resonance imaging, the sensor was temporarily removed.

Within the IGS software, individual vital sign trends were visualized, and complementary to the hospital MEWS protocol, a partial MEWS (D-EWS) was presented every hour to promote adequate detection. The D-EWS was based on the HR and ReR measurements and was in line with the MEWS thresholds and scores (Multimedia Appendix 4) on the preinstalled thresholds for HR and ReR. Patient numbers and names were automatically synchronized with the EHR using a Health Level 7 linkage, so manual entry was not required. As the device measures only 2 vital signs, routine manual measurements of other relevant vital signs (eg, temperature and blood oxygen saturation) by nurses were maintained throughout the study. Every 4 hours (ie, twice per shift), nurses routinely assessed vital sign trends and reported the D-EWS and any deviations and subsequent nursing activities in the EHR at the end of every shift. When the D-EWS was ≥3, additional checks and interventions could be performed as deemed appropriate by the nurse. No alarm strategy was applied in this study based on the substantial alarm fatigue experienced by nurses in our previous feasibility studies [15,23].

### Implementation Strategy

Before the start of the study, the 2 wards were technically prepared for CMVS, and an e-learning module was developed (Textbox 1). This comprehensive 30-minute e-learning module (*Articulate 360; Articulate Global*) was developed by the project manager (JL) together with an educationalist. The e-learning module ended with a knowledge assessment that had to be successfully completed. The contents were pilot-tested by 4 nurses of the project team (Multimedia Appendix 5). This project team was formed per ward comprising the project manager, 4 nurses, the ward manager, and 1 consulting physician. First, information and goals about the project were presented in a regular team meeting 2 months before the start of the project. Subsequently, the e-learning module for nurses was made available on the web (Multimedia Appendix 5). It consisted of information about the purpose of the project, the rationale for CMVS, the protocol of the D-EWS, the work processes and policy, the practical use of the IGS system, and how to assess the vital sign trends. Afterward, there was a week of daily meetings with the project manager to provide ample opportunities to ask further questions. In addition, all relevant physicians were informed of the project and workflow in a team meeting.

During the first 4 weeks of the study, bedside training and coaching were provided by the project manager (Monday-Friday) 3 times daily. In addition, weekly status updates and feedback on the implementation were provided to the entire team via email. During the study period, the project manager coordinated the inclusion process (Monday-Friday). A small number of dedicated project team nurses acted as key users to provide support for all nurses.

To accurately monitor the implementation, the use of performance feedback was deemed essential. Each month of the study—as a structured evaluation moment—a dashboard with interim results of the inclusion rate and intervention fidelity was discussed in a project team meeting. In addition, a patient case study with deviating vital sign trends was presented, and CMVS experiences were discussed. Subsequently, actions were defined according to the Plan Do Check Act cycle [37], resulting in an iterative process of improvement of the implementation strategy. The results of the meeting, including the dashboard and related actions, were communicated to all team members via email. In addition, every 100th patient with CMVS was celebrated as an inclusion milestone in the team meeting.

**Planning of the implementation process.**

**Preparation period**

- Month –4
  Technical preparation of the ward
  Development of an e-learning module
- Month –3
  Plenary team meeting
- Month –2
  e-learning module on the web
  Defining implementation measures with key users
- Month –1
  Daily meetings for nurses
  Education for physicians

**Implementation period**

- Month 1
  Go live
  Bedside coaching 3 times a day
  Weekly feedback updates
  Monthly evaluation and feedback
- Months 2 to 6
  Monthly evaluation and feedback

### Study Procedures

Admitted patients who met the inclusion criteria were approached and received information about the study. Patients of surgery were asked for informed consent during the preadmission call, and patients of internal medicine were asked when admitted to the ward. When the patients agreed to participate, the nurse started CMVS directly or immediately after the surgical procedure until discharge.

### Sample Size

The study sample size was based on the primary aim of the project: evaluation of the implementation.

There is insufficient guidance in the literature regarding sample size calculations for this type of implementation evaluation studies. On the basis of historical data and the recruitment rate of our previous feasibility studies [15,23], we estimated that we would be able to include 350 patients across both wards over a period of 6 months.

### Ethical Considerations and Informed Consent

The Medical Ethics Committee of the Isala Hospital reviewed the protocol (protocol 210414) and declared that the Medical Research Involving Human Subjects Act (also known by its Dutch abbreviation WMO) did not apply for this study. This study was conducted in accordance with the Declaration of Helsinki. Written informed consent was obtained from all patients participating in the study. All patient data were registered in case report forms and stored securely.

### Data Collection

#### Quantitative Data

On the basis of the outcome definitions by Proctor et al [38], a broad range of implementation outcomes was assessed—overall and per ward—to comprehend the full extent of the implementation. An overview of the measured constructs and timing is presented in Table 1.

**Table 1:** Overview of study outcomes per ward.

Outcome	Month					
	1	2	3	4	5	6
Intervention fidelity	AD^a^	AD	AD	AD	AD	AD or I^b^
Implementation fidelity	AD	AD	AD	AD	AD	AD or I
Technical fidelity	AD	AD	AD	AD	AD	AD or I
Appropriateness	S^c^	N/A^d^	N/A	N/A	N/A	N/A
Acceptability	N/A	N/A	S	N/A	N/A	S/I
Usability	N/A	N/A	S	N/A	N/A	S/I
Adoption	N/A	N/A	S	N/A	N/A	N/A
Feasibility	N/A	N/A	S	N/A	N/A	S/I

^a^AD: administrative data from the patient record.

^b^I: interview.

^c^S: survey collected via email.

^d^N/A: not applicable.

The main outcome was the “intervention fidelity,” defined as the proportion of written nurse EHR reports on the CMVS trend assessment per patient per nursing shift. A 100% score would be 3 reports per 24 hours per patient. We considered 70% of written reports per patient to be sufficient for implementation success based on our previous feasibility study [15]. In addition, any follow-up nursing activities in case of deviating trends were described.

The secondary outcomes were implementation fidelity; technical fidelity; and a survey of nurses on appropriateness, acceptability, usability, adoption, and feasibility. For implementation fidelity, the proportion of nurses who had completed the e-learning module, the proportion of monthly evaluations with the project team, and the implementation measures were documented and described. In addition, exposure (defined as the proportion of hospitalized patients receiving the intervention at the ward during implementation), recruitment (defined as the proportion of actual patients willing to participate), and retention rate (defined as retention of patients using CMVS during admission) were recorded. Moreover, regarding technical fidelity, the following data of the CMVS system were collected: number of measurements, proportion of data artifacts, D-EWSs, and premature replacement of the sensor because of technical failure. An artifact was defined as an invalid measurement as identified by the algorithm of the system and presented as *-?-*.

The surveys for nurses were sent via email and consisted of several questionnaires (Table 1). The 4-item Acceptability of Intervention Measure evaluated acceptability, the 4-item Intervention Appropriateness Measure evaluated appropriateness, and the 4-item Feasibility of Intervention Measure evaluated feasibility, all on a 5-point Likert scale (score 1-5). A median score of ≥3.5 was considered sufficient [39].

The 10-item System Usability Scale measured usability on a 5-point Likert scale, resulting in a score of 0 to 100. A usability score of ≤50 was considered unacceptable, 51 to 70 was considered marginal, and >70 was considered acceptable [40].

The 15-item Evidence-Based Practice Attitude Scale on a 5-point Likert scale measured adoption (score of 0-4) with the following subscales: requirements, appeal, openness, and divergence. Scores were reported as overall scores and per subscale. A higher score indicated better adoption. A median score of ≥2.5 was defined as sufficient adoption.

Finally, we collected data on patient characteristics (gender, age, BMI, American Society of Anesthesiologists classification, urgency of admission, Short Nutritional Assessment Questionnaire [41], smoking status, alcohol use, and comorbidities [Charlson Comorbidity Index score ranging from 0-12]) [42,43] and outcomes (length of stay, mortality, unplanned intensive care unit admissions, and rapid response teams) from administrative data from the EHR. Nurse demographics (gender, age, job position, working experience in years, and working hours per week) were collected from the hospital’s personnel records.

#### Qualitative Data

In addition to the quantitative data, semistructured interviews were conducted with nurses (Table 1). The qualitative element of this study aimed to clarify the quantitative data. A pilot-tested topic list guided the interviews (Multimedia Appendix 6), which were conducted by 2 nursing students who were trained and supervised by the project manager (JL). The interviews were audio recorded and transcribed verbatim. No field notes were taken. Per ward, at least 10 semistructured interviews were conducted in a secluded room on the ward in the last month of the study.

### Data Analysis

#### Quantitative Data

Data were analyzed using descriptive and inductive statistics with SPSS Statistics (version 26; IBM Corp) for Windows. Each continuous parameter was checked for normality using the Kolmogorov-Smirnov test and visually using a *Q*-*Q* plot and histogram. Normality-based reporting was performed using means with SDs or medians with IQRs. For categorical data, frequencies and percentages were reported.

To explore the differences between the wards, the unpaired *t* test, Mann-Whitney *U* test, and chi-square test of the Fisher exact test were performed based on normality and test assumptions. In addition, multiple linear regression was performed for explorative analysis of intervention fidelity of the nurses based on the reports. The independent variables were the Charlson Comorbidity Index [42], length of stay, number of D-EWSs of ≥3, amount of artifact data (in percentage), and the month of implementation. Implementation month was a dummy variable divided into early (months 1-2), mid- (months 3-4), and late (months 5-6) implementation. For all tests, *P*<.05 was considered statistically significant.

#### Qualitative Data

The interviews were analyzed using deductive thematic analysis with the qualitative data analysis software NVivo (version 12; *QSR International*). The raw data were analyzed using a 6-stage thematic analysis as outlined by Braun and Clarke [44]. The stages included (1) immersion, (2) generating initial codes, (3) searching for and identifying themes, (4) reviewing themes, (5) defining and naming themes, and (6) writing the report.

In total, 2 researchers (JL and HR) conducted stages 1 to 3 independently. During the first and second stages, JL and HR became familiar with the data by listening to the audio recordings, checking the transcriptions against the audio recording, reading, listening to sections again, and rereading the final transcripts. During the third stage, both researchers read the transcripts and codes for categorizing similar statements into first themes. In stages 4 to 6, all authors were responsible for reviewing, defining, and naming themes through discussion. During the sixth stage, the themes were brought to the nurses for member checking via email, which did not result in any changes to the themes.

### Mixed Methods: Integration and Interpretation

Integration of the quantitative and qualitative elements of the study occurred through linking the methods of data collection and analysis. Linking of methods occurred through building: the data of the inclusion and intervention fidelity per month served as the start for the interview, and possible explanations based on the nurses’ experiences were discussed. Interpretation and reporting occurred through the contiguous approach: presentation of qualitative and quantitative findings in consequent but different sections [45].

## Results

### Study Characteristics

A total of 384 patients were screened for participation, of whom 6 (1.6%) declined. Of the 378 patients included during the implementation period, 20 (5.3%) were excluded because of conversion to palliative surgery (n=5, 25%), known allergy (n=1, 5%), loss to follow-up (n=8, 40%), surgery cancellation (n=3, 15%), retraction (n=2, 10%), or postoperative admission to another ward (n=1, 5%). Finally, 358 patients were included in the analysis: 248 (69.3%) from the surgical ward and 110 (30.7%) from the internal medicine ward (Multimedia Appendix 7). The median length of stay at the surgical ward was 6.0 (IQR 3.5-10.5) days versus 8.8 (IQR 5.5-14.1) days at the internal medicine ward (*P*<.001). Nearly all patients of internal medicine (109/110, 99.1%) were emergency admissions in contrast to 7.3% (18/248) at the surgical ward (*P*<.001), and in-hospital mortality was considerably higher in the internal medicine ward (8/110, 7.3% vs 2/248, 0.8%; *P*=.002). For all the characteristics, see Table 2. In total, 148 nurses participated in the study: 71 (48%) from the surgical ward and 77 (52%) from the internal ward (Table 3). The median age of the nurses was 29 (IQR 24-42) years; they were predominantly female (136/148, 91.9%), and 37.2% (55/148) were senior nurses. The median work experience was 5 (IQR 2-16) years, with a median of 32 (IQR 24-32) working hours per week. There were no significant differences between the characteristics of the 2 wards (Table 3).

**Table 2:** Study characteristics (n=358).

Characteristics		Surgery (n=248)	Internal medicine (n=110)	*P* value	
**Gender, n (%)**					.01^a,^^b^
	Man	138 (55.6)	77 (70)		
	Woman	110 (44.4)	33 (30)		
	Nonbinary	0 (0)	0 (0)		
Age (years), mean (SD)		67.8 (12.5)	71.2 (12.3)	.01^b,c^	
BMI (kg/m^2^), mean (SD)		26.4 (4.8)	28.5 (6.8)	.003^b,c^	
Length of stay (days), median (IQR)		6.0 (3.5-10.5)	8.8 (5.5-14.1)	<.001^b^^,^^d^	
Charlson Comorbidity Index, mean (SD)		5.0 (2.5)	4.0 (1.9)	<.001^a,^^b^	
**ASA^e^, n (%)**					<.001^a,^^b^
	1-2	141 (56.9)	27 (24.5)		
	3-4	107 (43.1)	55 (50)		
	Unknown	0 (0)	28 (25.5)		
**Urgency, n (%)**					<.001^f^
	Elective	230 (92.7)	1 (0.9)		
	Urgent	18 (7.3)	109 (99.1)		
**SNAQ^g^ score, n (%)**					.99^a^
	0-2	214 (86.3)	95 (86.4)		
	3-5	34 (13.7)	15 (13.6)		
**Katz-ADL^h^ score, n (%)**					<.001^a^
	0	214 (86.3)	72 (65.5)		
	1-6	34 (13.7)	38 (34.5)		
**Smoking, n (%)**					.34^a^
	Yes	39 (15.7)	18 (16.4)		
	No	80 (32.3)	49 (44.5)		
	Prior	129 (52)	43 (39.1)		
Alcohol (current use), n (%)		123 (49.6)	47 (42.7)	.23^a^	
Mortality, n (%)		2 (0.8)	8 (7.3)	.002^b,f^	
RRT^i^ calls, n (%)		3 (1.2)	0 (0)	N/A^j^	
Unplanned ICU^k^ admissions, n (%)		5 (2)	5 (4.5)	.18^f^	

^a^Chi-square test.

^b^Significant with *P*<.05.

^c^Unpaired *t* test.

^d^Mann-Whitney *U* test.

^e^ASA: American Society of Anesthesiologists.

^f^Fisher exact test.

^g^SNAQ: Short Nutritional Assessment Questionnaire.

^h^Katz-ADL: Katz Activities of Daily Living.

^i^RRT: rapid response team.

^j^N/A: not applicable.

^k^ICU: intensive care unit.

**Table 3:** Characteristics of professionals (n=148).

Characteristics		Surgery (n=71)	Internal (n=77)	*P* value
Gender (man), n (%)		3 (4.2)	9 (11.7)	.10^a^
Age (years), median (IQR)		29 (25-44)	29 (24-41)	.99^b^
**Job position, n (%)**				.90^a^
	Nurse	45 (63.4)	48 (62.3)	
	Senior nurse	26 (36.6)	29 (37.7)	
Work experience (years), median (IQR)		5 (1-15)	5 (2-17.5)	.78^b^
Working hours per week, median (IQR)		32 (24-32)	32 (24-32)	.60^b^

^a^Chi-square test.

^b^Mann-Whitney *U* test.

### Intervention Fidelity

**Figure 1:**
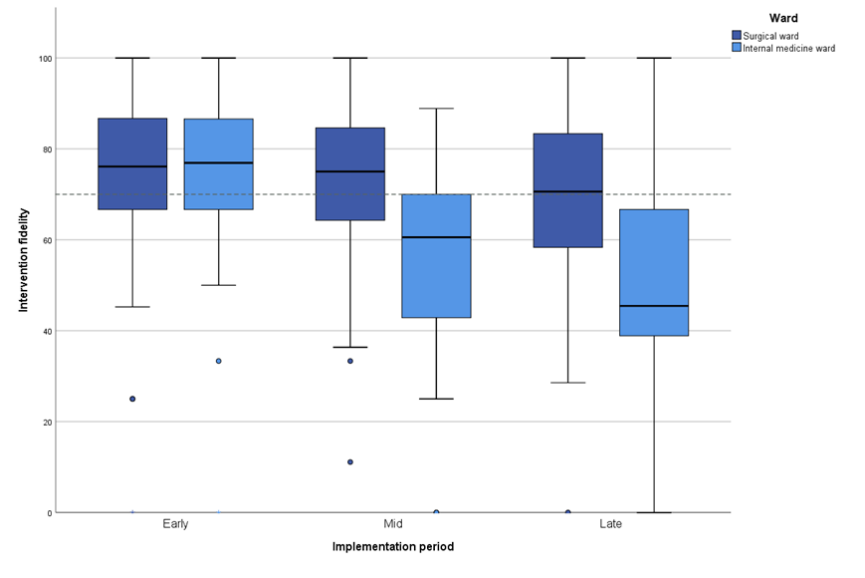
Intervention fidelity per ward over time. The dotted line represents the 70% threshold. Early: months 1 to 2; mid: months 3 to 4; late: months 5 to 6.

Eventually, 6142 shifts were analyzed. The overall mean intervention fidelity for both wards was 70.7% (SD 20.4%); it was considered sufficient in the surgical ward but not in the internal medicine ward (73.6%, SD 18.1% vs 64.1%, SD 23.7%; *P*<.001). Multiple regression analysis showed that intervention fidelity remained stable over time in the surgical ward but decreased over time in the internal medicine ward (76.3% at early implementation vs 56.5% at midimplementation vs 48.2% at late implementation; *P*<.001; Figure 1 and Table 4). Changes in intervention fidelity could not be explained by other variables (Multimedia Appendix 8).

With respect to the documented nursing activities (n=174; range 1-9 per patient), for most patients (246/358, 68.7%), no nursing activities were needed based on the vital sign trend assessments. A total of 101 interventions were carried out by nurses individually; it mostly consisted of an extra bedside assessment of the patient followed by wait and see (n=73). In addition, 73 activities were performed after consultation with a physician (59/73, 81% of these were at the surgical ward; Table 5).

**Table 4:** Intervention, implementation, and technical fidelity.

			Total	Surgery	Internal medicine	*P* value
**Intervention fidelity**						
	Written nurse reports, n (%)		6142 (100)	3134 (70.8)^a^	1153 (67.3)^b^	.008^c,d^
	Patients >70% threshold, n (%)		198 (55.3)^e^	150 (60.5)^f^	48 (43.6)^g^	.003^c,d^
	**Overall fidelity (%), mean (SD)**		70.7 (20.4)	73.6 (18.1)	64.1 (23.7)	<.001^c,h^
		Early implementation (months 1-2)	75.8 (17.2)	75.6 (17.2)^i^	76.3 (17.4)^j^	.80^h^
		Midimplementation (months 3-4)	67.4 (21.4)	73.8 (18.5)^k^	56.5 (21.8)^l^	<.001^c,h^
		Late implementation (months 5-6)	65.9 (22.3)	70.7 (18.8)^m^	48.2 (25.9)^n^	<.001^c,h^
	Recruitment rate, n (%)		358 (98.3)^o^	248 (98.4)^p^	110 (98.2)^q^	>.99^r^
	Retention rate, n (%)		358 (99.4)^s^	248 (99.2)^t^	110 (100)^u^	>.99
**Implementation fidelity**						
	Nurses who attended e-learning module, n (%)		147 (93)^v^	60 (89.6)^w^	87 (95.6)^x^	.21^r^
	Monthly evaluations, n (%)		10 (100)	5 (100)	5 (100)	N/A^y^
	Exposure, n (%)		358 (28)^z^	248 (33.6)^aa^	110 (21.8)^ab^	<.001^c,d^
**Technical fidelity (n=340)**						
	Monitoring time (hours), median (IQR)		96.6 (47.6-163.6)	96.2 (47.5-164.9)	97.4 (47.3-157.8)	.60^ac^
	**Total measurements, N**		1,017,467	729,622	287,845	N/A
		HR^ad^ measurements, N	508,226	364,285	143,941	N/A
		HR measurement artifacts, n (%)	136,753 (26.9)	83,527 (22.9)	53,226 (36.9)	<.001^c,d^
		ReR^ae^ measurements, N	509,281	365,377	143,904	N/A
		ReR measurement artifacts, n (%)	74,785 (14.7)	51,758 (14.2)	23,027 (16.0)	.04^c,d^
	**D-EWS^af^, N**		32,730	24,267	8463	<.001^c,d^
		Score of 0, n (%)	6610 (20.2)	5500 (22.7)	1110 (13.1)	
		Score of 1-2, n (%)	24,385 (74.5)	17,849 (73.6)	6536 (77.2)	
		Score of ≥3, n (%)	1734 (5.3)	917 (3.8)	817 (9.6)	
	Sensors replaced, n (%)^e^		37 (10.3)	27 (10.9)	10 (9.1)	.71^d^

^a^N=4428.

^b^N=1714.

^c^Significant with *P*<.05.

^d^Chi-square test.

^e^N=358.

^f^N=248.

^g^N=110.

^h^Unpaired *t* test.

^i^N=104.

^j^N=51.

^k^N=65.

^l^N=38.

^m^N=79.

^n^N=21.

^o^N=364.

^p^N=252.

^q^N=112.

^r^Fisher exact test.

^s^N=360.

^t^N=250.

^u^N=110.

^v^N=158.

^w^N=67.

^x^N=91.

^y^N/A: not applicable.

^z^N=1242.

^aa^N=738.

^ab^N=504.

^ac^Mann-Whitney *U* test.

^ad^HR: heart rate.

^ae^ReR: respiratory rate.

^af^D-EWS: partial Modified Early Warning Score.

**Table 5:** Documented nursing activities in continuous monitoring of vital signs reports.

					Surgery (n=248)			Internal medicine (n=110)
No reports available, n (%)					0 (0)			5 (4.5)
No activities, n (%)					168 (67.7)			78 (70.9)
**Activities performed by a** **nurse, N**					75			26
	Assessment (wait and see), n (%)			61 (81.3)^a^			13 (50)^b^	
	Additional manual check measurement with MEWS^c^, n (%)			14 (18.7)^a^			13 (50)^b^	
**Activities performed in consultation with a physician, N**					59			14
	Consulted physician but wait and see, n (%)			1 (1.7)^d^			2 (14.3)^e^	
	**Diagnostics, n (%)**			18 (30.5)^d^			5 (35.7)^e^	
		Blood test—blood culture	5 (8.5)^d^			2 (14.3)^e^		
		Chest x-ray	4 (6.8)^d^			0 (0)^e^		
		Electrocardiogram	2 (3.4)^d^			2 (14.3)^e^		
		CT^f^ scan	3 (5.1)^d^			0 (0)^e^		
		Urine sediment	2 (3.4)^d^			1 (7.1)^e^		
		Blood test—arterial blood gas	1 (1.7)^d^			0 (0)^e^		
		COVID-19 PCR^g^ test	1 (1.7)^d^			0 (0)^e^		
	**Therapy, n (%)**			40 (67.8)^d^			7 (50)^e^	
		Analgesics	18 (30.5)^d^			0 (0)^e^		
		Oxygen administration	6 (10.2)^d^			2 (14.3)^e^		
		Bronchodilators	6 (10.2)^d^			0 (0)^e^		
		Fluid challenge	3 (5.1)^d^			0 (0)^e^		
		β-blockers	2 (3.4)^d^			1 (7.1)^e^		
		Diuretics	2 (3.4)^d^			2 (14.3)^e^		
		Breathing exercise	2 (3.4)^d^			1 (7.1)^e^		
		Digoxin	1 (1.7)^d^			0 (0)^e^		
		Antibiotics	0 (0)^d^			1 (7.1)^e^		

^a^N=75.

^b^N=26.

^c^MEWS: Modified Early Warning Score.

^d^N=59.

^e^N=14.

^f^CT: computer tomography.

^g^PCR: polymerase chain reaction.

### Implementation Fidelity

Regarding implementation fidelity, most nurses attended the e-learning module, all elements of the strategy were delivered, and monthly evaluations were performed. There were 27 implementation measures conducted but no major changes in the intervention itself (Table 4 and Multimedia Appendix 9). Furthermore, recruitment and retention rates were 98.4% and 99.4%, respectively, and did not significantly differ between the wards (Table 3). Exposure to the intervention was 33.6% (248/738) of patients at the surgical ward versus 21.8% (110/504) of patients at the internal medicine ward (*P*<.001). In addition, the proportion of patients who participated over time in the internal medicine ward was 46.4% (51/110) at early implementation, 34.5% (38/110) at midimplementation, and 19.1% (21/110) at late implementation.

### Technical Fidelity

Regarding technical fidelity, a total of 45,113 hours of monitoring was available (Table 3). The median monitoring time was 96 (IQR 48-163) hours per patient, resulting in 1,017,467 vital sign measurements. The monitoring data from 340 patients were successfully retrieved. There were artifacts in 26.91% (136,753/508,226) of the HR measurements and 14.68% (74,785/509,281) of the ReR measurements. HR artifacts were significantly higher in the internal medicine ward (53,226/143,904, 36.99% vs 83,527/364,285, 22.93%; *P*<.001) for unknown reasons. Of all the devices, 10.3% (37/358) were prematurely replaced owing to technical failure. A total of 32,730 D-EWSs were generated by the system, of which 5.3% (1,734/32,730) were ≥3. The distribution of scores was different for the 2 wards (Table 4; *P*<.001).

### Nurses’ Surveys

A total of 194 surveys were returned (Table 6). At the start of the study, surgical nurses found the intervention sufficiently appropriate in contrast to internal medicine nurses (median score 4.0 vs 3.1; *P*=.03). In addition, the overall attitude toward the adoption of new interventions was high (score of 3.5) in both wards (*P*=.82). Nurses in both wards found the intervention sufficiently acceptable during the study but not at the end (score of 3.5 vs 3.0; *P*=.02). Acceptability was significantly lower in the internal medicine ward at the end of the study (*P*=.02). Usability was rated as marginal in both wards at both measurement times. Feasibility was rated as sufficient but decreased at the end of the study (score of 4.0 vs 3.4; *P*=.002).

**Table 6:** Nurses’ survey^a^.

		Total	Surgery	Internal medicine	*P* value (wards)	*P* value (time)	
Appropriateness, median (IQR)		3.75 (3.0-4.00)	4.00 (4.00-5.00)	3.13 (2.31-4.00)	.03^b,c^	N/A^d^	
**Adoption, median (IQR)**		3.47 (3.33-3.73)	3.50 (3.33-3.68)	3.47 (3.27-3.3.73)	.82^b^	N/A	
	Openness	4.00 (3.75-4.00)	4.00 (3.75-4.00)	4.00 (3.75-4.00)	.54^b^		
	Divergence	3.00 (2.50-3.00)	3.00 (2.50-3.00)	2.75 (2.38-3.25)	.94^b^		
	Appeal	4.00 (3.75-4.00)	4.00 (3.75-4.00)	4.00 (3.50-4.00)	.28^b^		
	Requirements	3.67 (3.00-4.00)	4.00 (3.00-4.00)	3.67 (3.00-4.00)	.75^b^		
**Acceptability, median (IQR)**							.02^c^ (overall); .10 (surgery); .07 (internal)
	T_1_^e^	3.5 (2.75-4.00)	3.75 (3.00-4.00)	3.25 (2.25-3.75)	.08^b^		
	T_2_^f^	3.0 (2.25-3.75)	3.00 (2.44-4.00)	2.5 (2.00-3.25)	.02^b,c^		
**Usability** **, mean (SD)**							.79 (overall); .51 (surgery); .69 (internal)
	T_1_	60.4 (10.8)	62.0 (10.8)	58.6 (10.6)	.23^g^		
	T_2_	61.0 (13.0)	63.8 (10.7)	57.2 (15.1)	.049^c,g^		
**Feasibility** **, median (IQR)**							.002^c^ (overall); .02^c^ (surgery); .02^c^ (internal)
	T_1_	4.00 (3.5-4.0)	4.00 (3.75-4.50)	3.75 (3.13-4.00)	.08^b^		
	T_2_	3.38 (3.0-4.0)	3.75 (3.00-4.00)	3.00 (3.00-3.75)	.03^b,c^		

^a^Appropriateness, acceptability, and feasibility: Likert scale from 1=disagree to 5=agree. Adoption: Likert scale from 0=disagree to 4=agree. Usability: scale from 0 to 100 (score ≥68=sufficient).

^b^Mann-Whitney *U* test.

^c^Significant with *P*<.05.

^d^N/A: not applicable.

^e^T_1_: month 3.

^f^T_2_: month 6.

^g^Unpaired *t* test.

### Qualitative Data

#### Characteristics

A total of 21 semistructured interviews were conducted with a mean duration of 10.9 (SD 3.3) minutes. Of the 21 interviewees, 11 (52%) worked at the surgical ward, 8 (38%) were senior nurses, and 3 (14%) were male. In total, 5 themes were identified.

#### Theme 1: Prioritizing CMVS

Nurses indicated that the prioritization of CMVS depended on the caseload during the shift. A commonly mentioned factor was perceived workload, frequently mentioned as tasks that they must perform during their shift. A nurse said the following:

Yes, I think it is when the workload is high, and then it easily forgotten because of it is not your priority to check and report the trend. If it’s just a quiet shift, then it’s easier to perform.

Internal medicine nurse 2

In addition, some nurses indicated that this varied by type of shift. Day shifts had a higher workload than evening and night shifts. Although night shifts were predominantly experienced as quieter, the actual intervention fidelity was not better during evenings or nights. A nurse explained the following:

During night shifts, I do not assess the vital signs trends because patients are supposed to be asleep and the standard manual measurement rounds are enough to assess their condition properly.

Internal medicine nurse 5

Nurses also experienced CMVS as a relatively unnecessary addition to their manual measurements, especially during the morning rounds, when the priority for additional trend assessments was lower. A nurse said the following:

Because in the morning you still measure your vital signs with the spot-check monitoring and then CMVS is on top of that. I am able to perform without those trends.

Surgical nurse 9

Furthermore, they indicated that, if the patients had an uncomplicated course, the direct need for assessing the vital sign trends was also considered less, and thus, regular trend assessments were less likely to be performed. However, when deemed clinically relevant, for instance, when the patient already had deviating vital signs or complications, they indicated that the correct assessment of trends was performed better. A nurse said the following:

If I only just once had a case where you can actually see deviating trends, then you’ll probably use CMVS better. My experience is (mainly) with stable patients who have CMVS that shows the same (stable) trends over three consecutive shifts; I think in that case actual use and usefulness fades a bit.

Internal medicine nurse 9

#### Theme 2: The Importance of a Bedside Nursing Assessment

Related to the priority of CMVS in the previous section, nurses mentioned the importance of their clinical bedside assessment. During routine morning rounds, vital sign measurements allow for the assessment of other dimensions besides vital signs, such as skin color, presence of sweating, dyspnea, and mental status and pain symptoms. In addition, other dimensions of nursing care can be assessed, such as checks on infusion therapy and excretion, but the need for physical care and care needs for upcoming discharge could also be inventoried through patient interaction. A nurse said the following:

During rounds we assess more than just measuring the values of the vital signs. For instance, in patients with oxygen supplementation, you really want to know what that the breathing looks like. ... Besides, by talking to the patient you can also obtain a more comprehensive impression of the patient who is lying in bed.

Internal medicine nurse 8

Many nurses found CMVS an addition to and sometimes support for their nursing work. Several said that trends were often a confirmation of their clinical perspective of the patient rather than it prompting them to reconsider their assessment. This was well reflected in the following statement:

I do find that when a patient is more ill, you assess the CMVS more often. ... But I do not often experience that it really detects something I did not know yet. ... However, I think it’s a very nice addition to our work and may possibly stimulate clinical reasoning; especially for young nurses.

Surgical nurse 7

In addition, a few nurses indicated that they did not yet fully trust the accuracy of the technology without physically assessing the patient. They indicated that they regularly found discrepancies between what they observed and what the trend indicated in ReR in particular. A nurse said the following:

And you have to compare trends to the patient context. For instance, with the respiratory rate. You have to verify if the patient is mobilized and assess if the trend deviation is clinically relevant.

Surgical nurse 9

#### Theme 3: Experiencing CMVS as an Added Value for Patient Care

Nurses differed in their opinions regarding the benefits for patient care of the intervention. Nurses who were positive about the added value of CMVS mentioned that it provided more insights into the patient’s clinical status, especially during night shifts and in patients who are critically ill. However, they also indicated that these types of patients do not often present at the general ward. In addition, several nurses mentioned that they had limited experience with the intervention and even no experience with deviating vital sign trends and taking action on them. Therefore, nurses questioned whether proactive trend assessment was feasible as standard care as, in many cases, it did not alter their nursing care at that time. A nurse said the following about this:

You have to assess regularly with most of the time not performing any actions based up on the trends. In my opinion, this does not bring any benefit to the patient, nor to us as professionals.

Internal medicine nurse 2

However, some nurses mentioned that, when they had witnessed a deviating trend and taken action as a result, the added value of the intervention had become clearer afterward. A nurse said the following:

I had a patient during my night shift with deviating trends, so I did an extra check and administered additional pain medication.

Surgical nurse 8

#### Theme 4: Experienced Usability of the CMVS System

The nurses frequently mentioned the experienced usability of the hardware and software as an explanation for the decreasing intervention fidelity. Although some nurses found that the necessary time investment was limited and CMVS was feasible during their shifts, several barriers to regular daily use were mentioned. The most often-mentioned barrier was the pairing of the sensor with the software platform as this had to be done through a separate web-based application on a prepared mobile phone rather than via the regularly used phone with a call system. A nurse said the following:

Sometimes the separate mobile phone with the specific codes malfunctions and it simply takes too much time, which eventually results in that you leave it at that.

Internal medicine nurse 5

Another barrier mentioned was the convenience of gaining visibility of the trends. The software for assessing trends was not integrated well enough into the EHR. Although the bed overview with patient names and numbers was paired, they preferred the trends to be also presented in the EHR or to be able to view them through a central monitoring display on the ward. Finally, removing the sensor when performing diagnostics for the prevention of interference was considered a barrier; in particular, they felt this was important as diagnostic tests such as electrocardiograms or computer tomography scans are often ordered for patients who are ill. A nurse said the following:

It is annoying when a sick patient has to go for a scan and then just at that important moment, the sensor must be removed.

Surgical nurse 4

#### Theme 5: Future Perspectives of CMVS on the General Ward

Several nurses shared their thoughts on what improvements are needed for future routine use. In addition to full integration of the software into the EHR, as mentioned in the previous section, nurses considered it important that the sensor be able to measure more vital signs than only HR and ReR. The main reason for this was that manual measurements of the other routine parameters (such as blood pressure and blood oxygen saturation) are still considered necessary, and therefore, CMVS with just HR and ReR does not result in measurable time-saving benefits. This would only be possible when all vital sign measurements and trends are directly visualized in the EHR. Although this would save time, it would not eliminate the need and value of bedside nursing assessments during rounds, as discussed in the previous section. A nurse said the following:

It would help enormously (all data and trends visible in the EHR), but even if everything is measured automatically, you still have to go and assess the patient yourself.

Surgical nurse 11

Another future perspective mentioned by some nurses was that specific alarm strategies for deviating trends could be an alternative to timely detect deterioration. However, they questioned whether the current MEWS is sensitive enough to detect many of the common complications where deviated vital sign values are not always present. A nurse said the following:

Yes I also hear my colleagues about it: when scoring a (MEWS of) 3 or higher, they do not perform repeat measurements because the respiratory rate is normal for this patient. ... I do think it’s sometimes way too sensitive for a lot of patients.

Internal medicine nurse 7

Furthermore, some nurses thought that there might be benefits in continuing the intervention after discharge from the hospital. A reason given for this was that remote clinical assessment is more difficult in a home situation. Moreover, they found that it could potentially encourage early discharge by incorporating CMVS into an early recovery protocol, such as Enhanced Recovery After Surgery.

Finally, nurses indicated that the use of assistive technology is desirable for the future of nursing care, considering the enrichment of nursing care and in view of future challenges in terms of capacity shortages. A nurse said the following:

I do support the inclusion of technology and innovation in nursing care. I think we still integrate technology too little and therefore we are less familiar with it in nursing care. Support by technology can bring so much, and I think my colleagues sometimes forget that.

Internal medicine nurse 6

## Discussion

### Principal Findings

In this study, we evaluated the process of implementation of CMVS on 2 general wards. Using a comprehensive implementation strategy, our overall results suggest that CMVS was sufficiently implemented in both wards, although intervention fidelity was highly variable and decreased over time. This decrease was explained to a large extent by the declining intervention fidelity in the internal medicine ward (it remained stable in the surgical ward). Another contributing factor was that nurses in both wards perceived little added value to the intervention. Taken together, the results show the complexity and interconnectedness of implementation and intervention fidelity with the technology and the perceptions of nurses.

Although the recruitment and retention rates of the intervention were high, indicating high patient acceptance, both wards showed a decline in several dimensions of implementation: intervention fidelity (although not statistically significant for surgery), perceived acceptance, usability, and feasibility. Interestingly, this decline was lower in the surgical ward than in the internal medicine ward. There are several possible explanations for this discrepancy between the surgical and internal medicine wards. Although 110 patients were included in the internal medicine ward, compared with the surgical ward, exposure to the intervention was still limited (110/504, 21.8%) and decreased over time—especially during the last 2 months of implementation. Second, nurses in the internal medicine ward considered the intervention less relevant to their practice. A first possible explanation as far as the internal medicine department is concerned, is the hospitalization procedure for emergency patients. After presentation in the emergency room and subsequent admission to the acute ward for a maximum of 48 hours, the patient is then transferred to the internal medicine ward. At that time, the diagnosis is established, and treatment starts, and so these patients have already passed the precarious, critical stage of their condition, and deviations in vital signs may be considered of lower clinical relevance [46]. This was different in the surgical ward, where CMVS was started directly after surgery, the period in which the patient is at the highest risk of complications and deterioration [47]. This may also be an explanation for the low appropriateness ratings from nurses in the internal medicine ward. Nevertheless, it is noteworthy that the proportion of patients with abnormal D-EWSs was highest in the internal medicine ward, but this was not deemed clinically relevant.

Although a broad range of interventions was performed by nurses based on the trend assessments, several reasons might explain the perceived low added value of the intervention for nursing care. First, the rationale for using CMVS is likely to be less convincing when also maintaining the conventional manual nurse measurements to calculate the MEWS. This could be explained by the fact that nurses highly value being at the bedside and observing the patients themselves while performing their manual measurements. Nurses explained that they use this moment to perform a more comprehensive patient evaluation, including assessing domains of clinical deterioration other than vital signs as well as other nursing domains through patient interaction. Second, the high degree (246/358, 68.7% of all patients) to which no subsequent activities were initiated based on the trend analysis may indicate that intervention fidelity was limited for this reason. In general, nurses stated that they had little or no experience interpreting deviating vital sign trends. In specific cases, trends may have prompted more timely additional measurements or diagnostics, such as blood tests or imaging, or the initiation of a physician’s consultation, but overall, it remains difficult to determine to what extent vital sign trend monitoring actually contributed to decision-making.

In addition, the current state of the technology may have affected intervention fidelity. Despite the generation of a very large amount of data, technical difficulties remain. Approximately 10.3% (37/358) of the sensors had to be replaced prematurely owing to different types of failure, such as malfunctioning of the sensor during pairing, unexplainable sensor failure, or high artifact ratios in some patients. This was also reflected in the high artifact rate for HR measurements, which may have had a negative influence on intervention fidelity and acceptability. A possible explanation is that adequate HR measurements using an accelerometer may be more complicated, but this has not yet been adequately studied [35]. The higher HR artifact rate in the internal medicine ward is also unclear. We checked patients with high artifact rates for incorrect sensor placement, but this was rarely the cause. Current limitations of the technology likely contributed to the low usability scores during the implementation period in both wards. In the interviews, nurses commented that these issues made it cumbersome to use the system while reducing trust in the technology. Furthermore, the current threshold-based D-EWSs to guide trend assessment do not sufficiently consider the context of the patient (eg, “in bed” or “actively mobilizing”), resulting in contamination of vital sign trends (eg, simultaneously HR and ReR) that are actually normal as the patient is actively mobilizing. Consequently, it will be harder to recognize true deterioration early. In contrast, when nurses manually record an abnormal set of bedside vital signs, CMVS trends may show an important correlation with the current (abnormal) bedside observation and can support the nurse’s decision to seek consultation with the on-call physician. The correlation between vital signs and direct bedside observations is important for clinical decision-making, which is missing when relying entirely on remote trend assessments.

### Comparison With Other Work

Comparison of our results with those of previous studies is challenging because of differences in patient populations, monitoring devices, and outcomes addressed. Intervention fidelity in this study was somewhat lower than in our previous feasibility study over a period of 3 months with a similar CMVS intervention [15]—71% versus 81%, respectively. However, if we compare the first months, this difference is smaller (75.8% vs 80.5%).

The need to still perform manual vital sign measurements and the lack of experience in assessing deteriorating trend patterns—as previously mentioned by nurses—are likely to have affected nurses’ perspectives and may have influenced intervention fidelity. This observation is also in line with the results of our previous feasibility study [15]. Moreover, although abnormal HR and ReR are important signs of patient deterioration, evidence is still lacking that CMVS monitoring of only 2 vital signs is sufficient to capture most cases of deterioration. In contrast to our results, Verrillo et al [48] showed that, when CMVS using a bulkier multiparameter device was used as the single method for vital sign monitoring, nurses’ acceptance and compliance over a period of only 6 weeks increased (initially 38% to a sustained average of 62% compliance). This may indicate that automating the manual measurements is better for the acceptability of nurses. Nonetheless, larger devices measuring all vital signs may result in poorer patient acceptance. Early termination of the intervention was rare in our study, which is in contrast to the 21% of patients in a previous study with a wrist-worn multiparameter device [14]. However, in our study, approximately 10.3% (37/358) of the sensors were prematurely replaced owing to technical errors such as connectivity issues. Furthermore, the need to gain experience with the use of the wearable device in clinical practice was also mentioned by nurses in the study by Izmailova et al [49]. Moreover, in line with previous studies, nurses also sometimes questioned the accuracy of the device and doubted the benefits of being able to observe their patients’ vital signs remotely [50,51]. In contrast, many nurses also expressed a positive attitude toward CMVS interventions, mentioning that it could increase patient safety by providing more insight [52]. Finally, experienced usability of a similar wearable patch device in ward nurses was higher in the study by Boatin et al [53], although this may be because of the relatively small, short-term study of 32 pregnant women.

Other studies have also reported on technical fidelity. Our observed artifact rates were slightly higher compared with a validation study in patients of surgery at the postanesthesia care unit with the same sensor [35]. A potential explanation is that motion artifacts are more prevalent in patients in wards than in patients during the early stages of recovery after anesthesia and surgery in the postanesthesia care unit.

### Limitations

To our knowledge, this is the first study that extensively focused on evaluating the process of CMVS implementation at scale in daily clinical practice in general hospital wards. The data can provide valuable information to other hospitals considering CMVS implementation and highlight some important issues to consider when developing an implementation strategy. However, several limitations should be considered when interpreting the results. First, in both wards, exposure to the intervention was still limited, which forced nurses to work with 2 systems of vital sign monitoring (intermittent and continuous) and may have hampered implementation. Second, it is important to note that the development of the implementation strategy and intervention took place in the surgical ward, which might have resulted in an intervention more suited to a surgical ward than to an internal medicine ward. In addition, goodwill toward the project manager, a former nurse in the surgical ward, might partly explain the higher intervention fidelity in the surgical ward. Third, even after analyzing every individual nurse trend assessment report, it is still not possible to determine with certainty to what extent these vital sign trends actually influenced subsequent diagnostic or therapeutic decision or both. This is mainly because several other factors contribute to additional activities and medical decision-making. Moreover, it is not clear how large the variation is between nurses interpreting similar trends. This would require a separate study. Finally, we did not include the magnitude of nurses’ exposure to the intervention as a factor in the regression analysis, which could cause bias. However, we extensively focused on education and bedside training in the implementation strategy.

### Implications

Our study highlights the complexity of implementing a CMVS system with wearable wireless sensors in hospital nursing wards. Therefore, policy makers should involve nurses early in establishing the intervention and implementation strategy and selecting the appropriate patient populations to enhance the fit with the needs of current nursing practice. To leverage the full potential of CMVS in general wards, several barriers to implementation in the routine workflow need to be addressed, for which we suggest the recommendations outlined in Textbox 2.

**Recommendations to address the barriers to implementation in the routine workflow.**

- Secure full and seamless integration of the continuous monitoring of vital signs (CMVS) into the hospital electronic health record, avoiding any separate software platforms or dashboards. This will improve fidelity and usability for caregivers.
- Use advanced and validated multiparameter CMVS sensors, which are sufficiently accurate and comprehensive to allow for the discontinuation of standard manual vital sign measurements by nurses, thus reducing nursing workload.
- Combine CMVS with reliable personalized clinical decision support tools to facilitate correct and timely interpretation of these measurements. Algorithms still need to be developed that can incorporate patient-specific baseline data, facilitate routine automated input of contextual factors such as patient movement, and perform automated trend analysis and event detection to timely detect and alert on clinical deterioration [22]. When such systems are available, this will obviate the need for vital sign trends to be proactively monitored and interpreted by nurses, which currently increases nursing workload and is difficult because of their lack of experience in this respect.
- Finally, carefully select (high-risk) patient populations that are likely to benefit most from CMVS. This could potentially include all acute care admissions (especially those without a clear diagnosis at admission) and all patients undergoing intermediate- or high-risk surgery in the postoperative phase (both in the ward and at home directly after discharge). Thus, the intervention could be integrated into an early discharge protocol with extended telemonitoring at the patient’s home [54].

### Conclusions

We successfully implemented a system for continuous wearable remote vital sign monitoring at scale in 2 hospital wards, but our results show that intervention fidelity decreased over time, to a larger extent in the internal medicine ward than in the surgical ward. This decrease appears to be dependent on multiple ward-specific factors. Nurses’ perceptions regarding the value and benefits of the intervention were variable. Our study provides valuable insights into the optimal implementation of CMVS in general wards. Specifically, we conclude that implementation of a CMVS while at the same time maintaining routine manual vital sign measurements is not advisable as it increases nurse workload. Proactive vital sign trend assessment by nurses is feasible but challenging to embed sustainably at scale in current workflows even when using an extensive implementation strategy. Wearable wireless monitoring technology should be further developed and optimized, including seamless integration into the EHR and development of more sophisticated decision support tools for interpretation and alarms that are suitable for general wards, before it can consistently improve nursing workflows, increase patient safety, and enhance quality of care.

## Appendix

Multimedia Appendix 1 Admission indications of patients.

Multimedia Appendix 2 The Philips Healthdot wearable sensor.

Multimedia Appendix 3 Example of the Philips IntelliVue Guardian Solution dashboard.

Multimedia Appendix 4 Thresholds of the partial Modified Early Warning Scores.

Multimedia Appendix 5 Contents and study goals of the e-learning module for nurses.

Multimedia Appendix 6 Topic list for the semistructured interviews with nurses.

Multimedia Appendix 7 List of admission indications.

Multimedia Appendix 8 Outcomes of multiple linear regression of intervention fidelity for both wards.

Multimedia Appendix 9 List of implementation measures of the monthly evaluations.

## Abbreviations

CMVS: continuous monitoring of vital signs
D-EWS: partial Modified Early Warning Score
EHR: electronic health record
EWS: Early Warning Score
HR: heart rate
IGS: IntelliVue Guardian Solution
MEWS: Modified Early Warning Score
ReR: respiratory rate
