# Supplementary material for: Process Evaluation of a Wireless Wearable Continuous Vital Signs Monitoring Intervention in 2 General Hospital Wards: Mixed Methods Study
Source: JMIR Nurs. 2023 May 4;6:e44061. doi: 10.2196/44061 (PMC10196902; doi:10.2196/44061)
Supplement: Multimedia Appendix 1 [file nursing_v6i1e44061_app1.docx]

**MULTIMEDIA APPENDIX 1: Admission indications of patients**

| Surgical ward | | Internal ward | |
| --- | --- | --- | --- |
| Gastro intestinal | Colorectal resections  Liver resections  Pancreatic | Gastroenterology | Pancreatitis  Acute gastro-intestinal bleeds  Liver cirrhosis |
| Vascular | Diabetic feet  Abdominal aortic aneurysm  Peripheral arterial disease (Fontaine III or IV) | General | Erysipelas  Pneumonia  Urinary tract infection  Multiple organ disorders |

This is a Multimedia Appendix to a full manuscript published in the J Med Internet Res. For full copyright and citation information see http://dx.doi.org/10.2196/jmir.44061
