# Supplementary material for: Process Evaluation of a Wireless Wearable Continuous Vital Signs Monitoring Intervention in 2 General Hospital Wards: Mixed Methods Study
Source: JMIR Nurs. 2023 May 4;6:e44061. doi: 10.2196/44061 (PMC10196902; doi:10.2196/44061)
Supplement: Multimedia Appendix 2 [file nursing_v6i1e44061_app2.docx]

**MULTIMEDIA APPENDIX 2: The Philips Healthdot wearable sensor**


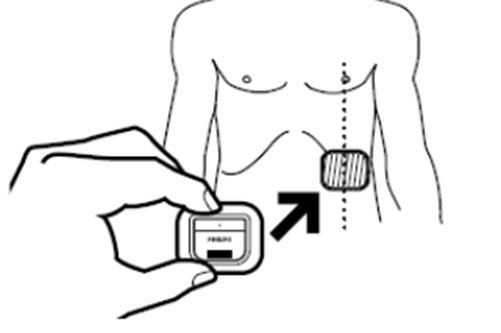


Reprinted from Philips Electronic Nederland BV under a CC BY license, with permission from Philips Electronic Nederland BV, original copyright 2020

This is a Multimedia Appendix to a full manuscript published in the J Med Internet Res. For full copyright and citation information see http://dx.doi.org/10.2196/jmir.44061
