# Supplementary material for: Process Evaluation of a Wireless Wearable Continuous Vital Signs Monitoring Intervention in 2 General Hospital Wards: Mixed Methods Study
Source: JMIR Nurs. 2023 May 4;6:e44061. doi: 10.2196/44061 (PMC10196902; doi:10.2196/44061)
Supplement: Multimedia Appendix 3 [file nursing_v6i1e44061_app3.pdf]

MULTIMEDIA APPENDIX 3: Example of the Philips Intellivue Guardian Solution (IGS) dashboard

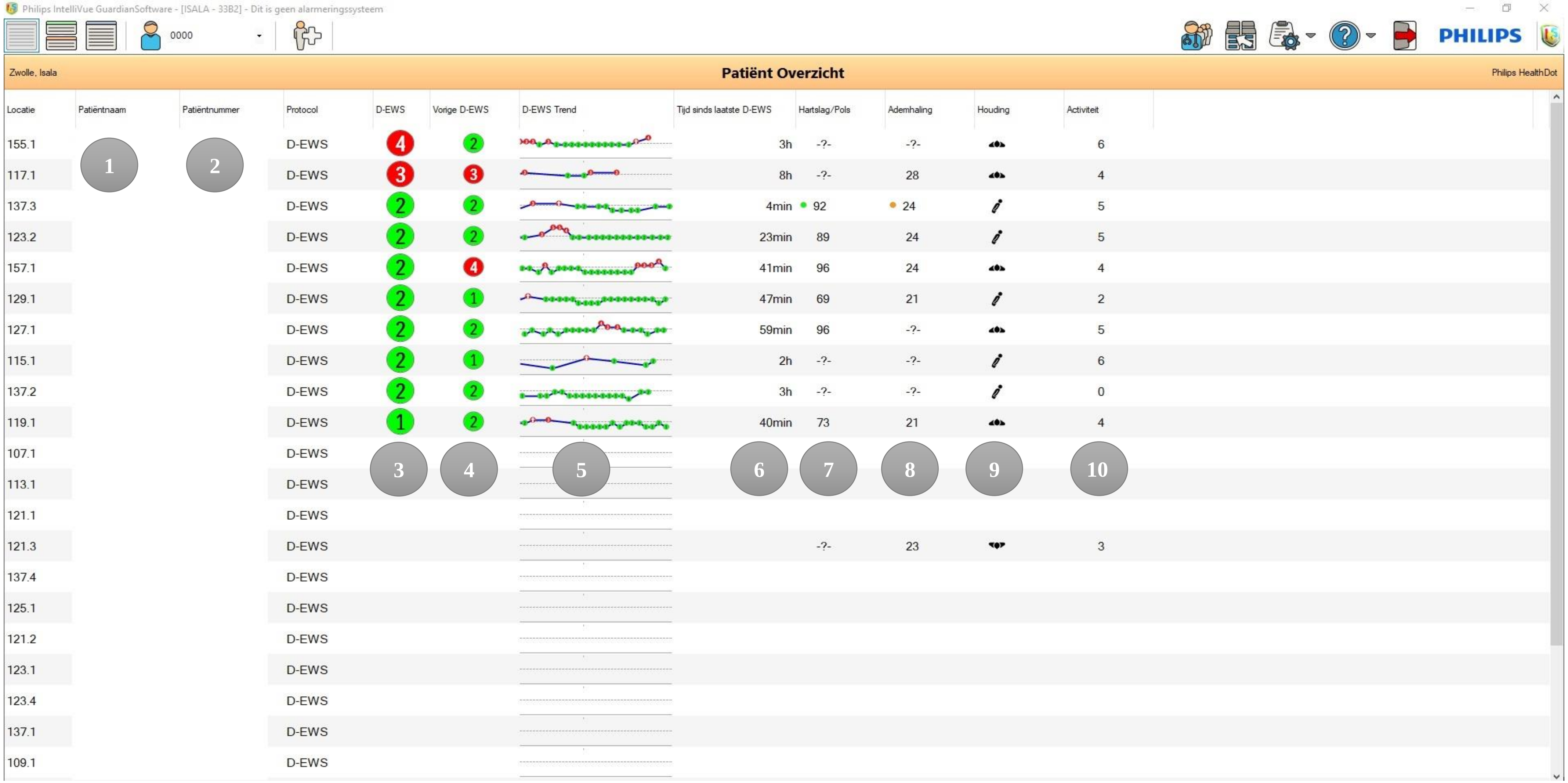

1: Patient’s name, 2: Patient ID, 3: latest D-EWS score, 4: previous D-EWS score, 5: trend of D-EWS scores over a period of 24 hours, 6: Time since latest D-EWS score, 7: latest heartrate measurement, 8: latest respiratory rate measurement, 9: latest posture measurement, 10: latest activity score

This is a Multimedia Appendix to a full manuscript published in the J Med Internet Res. For full copyright and citation information see http://dx.doi.org/10.2196/jmir.44061
