# Supplementary material for: Process Evaluation of a Wireless Wearable Continuous Vital Signs Monitoring Intervention in 2 General Hospital Wards: Mixed Methods Study
Source: JMIR Nurs. 2023 May 4;6:e44061. doi: 10.2196/44061 (PMC10196902; doi:10.2196/44061)
Supplement: Multimedia Appendix 4 [file nursing_v6i1e44061_app4.docx]

**MULTIMEDIA APPENDIX 4: Thresholds of the partial Early Warning (D-EWS) scores**

|  | **D-EWS score** | | | | | |
| --- | --- | --- | --- | --- | --- | --- |
| Score | 2 | 1 | 0 | 1 | 2 | 3 |
| Heart rate (HR) | <40 | 40-50 | 51-100 | 101-110 | 111-130 | >130 |
| Respiratory rate (ReR) | <9 |  | 9-14 | 15-20 | 21-30 | >30 |

This is a Multimedia Appendix to a full manuscript published in the J Med Internet Res. For full copyright and citation information see http://dx.doi.org/10.2196/jmir.44061
