# Supplementary material for: Process Evaluation of a Wireless Wearable Continuous Vital Signs Monitoring Intervention in 2 General Hospital Wards: Mixed Methods Study
Source: JMIR Nurs. 2023 May 4;6:e44061. doi: 10.2196/44061 (PMC10196902; doi:10.2196/44061)
Supplement: Multimedia Appendix 5 [file nursing_v6i1e44061_app5.docx]

**MULTIMEDIA APPENDIX 5: Contents and study goals of the e-learning for nurses**

**Contents**

- 1. About wearable continuous vital signs monitoring on the ward
  2. About the wearable sensor and nationwide developments
  3. About the project in Isala
  4. Getting started with continuous vital signs monitoring

4.1 The D-EWS protocol

4.2. Inclusion phase

Activation and applying the wearable sensor

Connecting the wearable sensor to the EHR

- 1. Monitoring phase

Assessments of vital signs trends

4.4. Closing phase

- 1. Guardian software

5.1 About the software

5.2 The software at your desktop

5.3 The software at your mobile application

- 1. Assessment

**Study goals**

1. You understand the rationale for using the continuous vitals sign monitoring and you are familiar with the project design.
2. You understand which and how vital signs are measured by the continuous vitals sign monitoring.
3. You understand how to include the patient and how to counsel patients about continuous vitals sign monitoring.
4. You are able to activate the sensor, pair it to the software and attach it on the patient's body at the right location.
5. You are able to manually add a new patient to the software. You can link the continuous vitals sign monitoring to the right patient via the app.
6. You are familiar with the two methods (at your desktop and mobile device) to display the data of the continuous vitals sign monitoring.
7. You are familiar with how the measurements of the continuous vitals sign monitoring are shown.
8. You understand when to assess the trend and what the follow-up interventions of deterioration trends should be.
9. You understand how to assess vital signs trends properly.
10. You are able to discharge the patient from the continuous vital sign monitoring.
11. You understand how you and your ward are supported during the project.

This is a Multimedia Appendix to a full manuscript published in the J Med Internet Res. For full copyright and citation information see http://dx.doi.org/10.2196/jmir.44061
