# Supplementary material for: Process Evaluation of a Wireless Wearable Continuous Vital Signs Monitoring Intervention in 2 General Hospital Wards: Mixed Methods Study
Source: JMIR Nurs. 2023 May 4;6:e44061. doi: 10.2196/44061 (PMC10196902; doi:10.2196/44061)
Supplement: Multimedia Appendix 6 [file nursing_v6i1e44061_app6.docx]

**MULTIMEDIA APPENDIX 6: Topic list for the semi-structured interviews with nurses**

| Introduction | Presenting the dashboard of the study with the monthly inclusion and intervention fidelity of the ward |
| --- | --- |
| 1 | Considering these interim results, what was your overall experience of working with the intervention? |
| 2 | Could you clarify these results when considering your experience? |
| 3 | To what extent does working with the intervention improve your daily work? |
| 4 | What would be your recommendations for the future with regard to working with the intervention? |

This is a Multimedia Appendix to a full manuscript published in the J Med Internet Res. For full copyright and citation information see http://dx.doi.org/10.2196/jmir.44061
