# Supplementary material for: Process Evaluation of a Wireless Wearable Continuous Vital Signs Monitoring Intervention in 2 General Hospital Wards: Mixed Methods Study
Source: JMIR Nurs. 2023 May 4;6:e44061. doi: 10.2196/44061 (PMC10196902; doi:10.2196/44061)
Supplement: Multimedia Appendix 7 [file nursing_v6i1e44061_app7.docx]

| **MULTIMEDIA APPENDIX 7: List of Admission indications** | | | |
| --- | --- | --- | --- |
| **Surgical ward (n=248)** | | **Internal ward (n=110)** | |
| **Gastro-intestinal surgery (n=201)** | | **General internal medicine (n=56)** | |
| Colorectal resection | 126 (62.7) | Pneumonia | 21 (37.5) |
| Pancreatic resection | 36 (17.9) | Urinal tract infections | 13 (23.2) |
| Liver resection | 15 (7.5) | Infectious disease, other | 9 (16.1) |
| Anus praetor construction | 15 (7.5) | Multiple organ disorders | 8 (14.3) |
| Other | 9 (4.8) | Erysipelas | 5 (8.9) |
| **Vascular surgery (n=47)** | | **Gastroenterology (n=54)** | |
| Peripheral occlusion | 36 (76.6) | Pancreatitis | 21 (38.9) |
| Bypass | 14 (29.8) | Gastrointestinal bleed | 20 (37.0) |
| Endarterectomy | 13 (27.7) | Liver cirrhosis | 11 (20.4) |
| Percutaneous Transluminal  Angioplasty | 9 (19.1) | Other | 2 (3.7) |
| Central occlusion | 6 (12.8) |  |  |
| Amputation | 4 (8.5) |  |  |
| Other | 1 (2.1) |  |  |

This is a Multimedia Appendix to a full manuscript published in the J Med Internet Res. For full copyright and citation information see http://dx.doi.org/10.2196/jmir.4406
