# Supplementary material for: Process Evaluation of a Wireless Wearable Continuous Vital Signs Monitoring Intervention in 2 General Hospital Wards: Mixed Methods Study
Source: JMIR Nurs. 2023 May 4;6:e44061. doi: 10.2196/44061 (PMC10196902; doi:10.2196/44061)
Supplement: Multimedia Appendix 8 [file nursing_v6i1e44061_app8.docx]

**MULTIMEDIA APPENDIX 8: Outcomes of multiple linear regression for both wards**

**Surgical ward**

| **Coefficients** | | | | | | | | |
| --- | --- | --- | --- | --- | --- | --- | --- | --- |
| Model | | Unstandardized Coefficients | | Standardized Coefficients | t | Sig. | 95,0% Confidence Interval for B | |
|  |  | B | Std. Error | Beta |  |  | Lower Bound | Upper Bound |
| 1 | (Constant) | 79.703 | 3.145 |  | 25.344 | .000 | 73.508 | 85.897 |
|  | CCI score | -.038 | .612 | -.004 | -.062 | .951 | -1.243 | 1.168 |
|  | D-EWS scores ≥3 | -.094 | .114 | -.057 | -.821 | .412 | -.318 | .131 |
|  | Length of stay | -.289 | .162 | -.123 | -1.780 | .076 | -.609 | .031 |
|  | Artefact rate | -.064 | .079 | -.052 | -.811 | .418 | -.218 | .091 |
|  | Mid implementation  (month 3-4) | -1.690 | 2.903 | -.041 | -.582 | .561 | -7.408 | 4.028 |
|  | Late implementation  (month 5-6) | -4.902 | 2.698 | -.126 | -1.817 | .071 | -10.217 | .413 |
|  | | | | | | | | |

**Internal medicine ward**

| **Coefficients** | | | | | | | | |
| --- | --- | --- | --- | --- | --- | --- | --- | --- |
| Model | | Unstandardized Coefficients | | Standardized Coefficients | t | Sig. | 95,0% Confidence Interval for B | |
|  |  | B | Std. Error | Beta |  |  | Lower Bound | Upper Bound |
| 1 | (Constant) | 79.274 | 6.433 |  | 12.323 | .000 | 66.516 | 92.032 |
|  | CCI score | -.656 | 1.088 | -.052 | -.603 | .548 | -2.814 | 1.501 |
|  | D-EWS scores ≥3 | .023 | .110 | .018 | .207 | .837 | -.195 | .240 |
|  | Length of stay | .140 | .240 | .050 | .584 | .561 | -.335 | .615 |
|  | Artefact rate | -.065 | .106 | -.053 | -.618 | .538 | -.275 | .144 |
|  | Mid implementation  (month 3-4) | -20.200 | 4,604 | -.407 | -4.388 | .000 | -29.331 | -11.070 |
|  | Late implementation (month 5-6) | -28.616 | 5.498 | -.477 | -5.205 | .000 | -39.519 | -17.712 |
|  | | | | | | | | |

Abbreviations: CCI: Charlson Comorbidity Index

This is a Multimedia Appendix to a full manuscript published in the J Med Internet Res. For full copyright and citation information see http://dx.doi.org/10.2196/jmir.4406
