# Supplementary material for: Process Evaluation of a Wireless Wearable Continuous Vital Signs Monitoring Intervention in 2 General Hospital Wards: Mixed Methods Study
Source: JMIR Nurs. 2023 May 4;6:e44061. doi: 10.2196/44061 (PMC10196902; doi:10.2196/44061)
Supplement: Multimedia Appendix 9 [file nursing_v6i1e44061_app9.docx]

**MULTIMEDIA APPENDIX 9: List of implementation measures** **of the monthly evaluations**

| Month | Surgical ward | Internal ward |
| --- | --- | --- |
| 1 | Describing a deterioration case study and communicate this by monthly mail | Request a schedule from assistant physicians to proactively provide education |
|  | Creating a dashboard for nurses for the daily meeting | Daily calls by the project manager about the status inclusions again in the afternoon  Explanation of the added value of trend monitoring after the acute phase of illness in the monthly evaluation  Discussing trends of actual patients in the daily education meeting four times |
| 2 | Adjusting activity plan in the EMR to make it more visible | Maintaining daily contact with project manager about inclusion |
|  | Using the daily boards in day and evening shifts. For nights shifts in handover-file. | Informing physicians about the progress of the project |
|  | Identify 'stragglers' based on file research and providing feedback to these nurses by key users. | Discussing trends of actual patients in the daily education meeting four times |
|  | Organizing of four education moments in the coming month about deteriorating trends |  |
|  | Sending an e-mail to all physicians as reminder of the project |  |
| 3 | Analysing a case study of deviating trend of a patient unplanned admitted to the ICU | Weekly training during the daily meeting on the potential added value of CMVS and with case studies |
|  | Providing feedback by e-mail on use in night shifts and added value of CMVS | Planning a presentation about the project in the team meeting |
|  | Promoting of verbal handover of CMVS between day and evening shift | Maintaining daily contact with project manager about inclusion |
|  | Identification of reasons of non-compliance during night-shifts |  |
|  | Planning a presentation about the project in the team meeting |  |
| 4 | Informing new physician assistants | Informing new physician assistants |
|  | Generating an educational quiz for the daily educational meeting | Maintaining daily contact with project manager about inclusion |
|  | Increasing the standard time intervals of trend software to maximum of four days |  |
| 5 | Checking whether logging in to the app still works on the mobile device | Maintaining daily contact with project manager about inclusion |

This is a Multimedia Appendix to a full manuscript published in the J Med Internet Res. For full copyright and citation information see http://dx.doi.org/10.2196/jmir.44061
